# Overexpression of CyclinA2 ameliorates hypoxia-impaired proliferation of cardiomyocytes

**DOI:** 10.3892/etm.2014.1935

**Published:** 2014-08-26

**Authors:** HUILING DENG, YONG CHENG, ZHIKUN GUO, FENXI ZHANG, XING LU, LINGLING FENG, XIANWEI WANG, ZHENPING XU

**Affiliations:** 1 Department of Science and Technology, Xinxiang Medical University, Xinxiang, Henan 453003, P.R. China; 2 Heart Center of Zhengzhou Ninth People’s Hospital, Zhengzhou, Henan 450000, P.R. China; 3 Key Laboratory of Henan Province for Medical Tissue Regeneration, Xinxiang Medical University, Xinxiang, Henan 453003, P.R. China; 4 Department of Anatomy, Xinxiang Medical University, Xinxiang, Henan 453003, P.R. China

**Keywords:** CyclinA2, hypoxia, adenovirus transfection, cardiomyocytes, proliferation

## Abstract

Hypoxia is a primary mediator for cell survival, and has been reported to inhibit cardiomyocyte proliferation in fetal and neonatal hearts. CyclinA2 is a key regulator of cell proliferation. Whether CyclinA2 affects cardiomyocyte proliferation in hypoxic conditions remains unexamined. This study was designed to investigate the roles of CyclinA2 expression on hypoxia-impaired cardiomyocyte proliferation. Cardiomyocytes were isolated from neonatal rats and randomly separated into six groups: Control, hypoxia, enhanced green fluorescent protein (EGFP)-Adv, EGFP-Ccna2, EGFP-Adv + hypoxia and EGFP-Ccna2 + hypoxia. The cells in the control group were cultured in a general cell incubator; the cells in the hypoxia group were placed in a hypoxic chamber for 12 h; the cells in the EGFP-Adv and EGFP-Ccna2 groups were separately transfected with EGFP-adenovirus capsids or EGFP-adenovirus capsids with CyclinA2 cDNA for 18 h, and then placed in a general incubator for an additional 12 h; the cells in the EGFP-Adv + hypoxia and EGFP-Ccna2 + hypoxia groups were separately transfected with EGFP-adenovirus capsids or EGFP-adenovirus capsids with CyclinA2 cDNA for 18 h, and then placed in a hypoxia chamber for an additional 12 h. CyclinA2 expression was measured using immunochemical staining and western blot analysis, and cardiomyocyte proliferation was measured using the cell counting kit 8. GFP fluorescence indicated a high transfection efficiency (>80%), and immunochemical staining showed that CyclinA2 was mainly distributed in the nucleus. CyclinA2 expression was downregulated following exposure to hypoxia for 12 h. Cardiomyocyte proliferation was also significantly decreased following exposure to hypoxia for 12 h. However, compared with the EGFP-Adv group, CyclinA2 expression and cardiomyocyte proliferation was markedly increased in the EGFP-Ccna2 group. Furthermore, compared with the EGFP-Adv + hypoxia group, CyclinA2 expression and cell proliferation were markedly increased in the EGFP-Ccna2 + hypoxia group. These findings indicate that CyclinA2 upregulation improves cardiomyocyte proliferation in hypoxic conditions.

## Introduction

Heart failure (HF) following myocardial infarction is the leading cause of morbidity and mortality in the world. Coronary artery disease (CAD) is the most risk important factor for HF, particularly among the older populations (1). Myocardial ischemia occurs following CAD (2). The ischemic injury causes cardiomyocyte death and myocardial fibrosis (3). The loss of cardiomyocytes and accumulation of extracellular matrix (ECM) lead to myocardial stiffness, dysfunction and eventually failure (2,3).

Ischemia is a process of insufficient blood flow in tissues that results in hypoxia (low oxygen supply). Hypoxia is the most important stimulus that leads to cardiomyocyte death in the process of heart ischemia. Both *in vitro* and *in vivo* studies have demonstrated that hypoxia can induce apoptosis and inhibit proliferation in cardiomyocytes (4–6). Previous studies have demonstrated that hypoxia-inducible factor 1α (HIF-1α) and heat shock factor 60 (HSF60) are two important molecular determinants of cardiomyocyte apoptosis in response to myocardial ischemia/reperfusion and hypoxia (1,7,8). Both molecules are acutely and chronically expressed in myocardial cells in response to hypoxia and ischemia, and have diverse targets that affect cell survival (1). HIF-1α expression promotes cardiomyocyte apoptosis in response to hypoxia via regulating the transcription of B-cell lymphoma 2 and Bcl-associated X protein (Bax) (7). HSF60 forms a complex with Bax following the translocation of cytosolic HSF60 to the membrane and Bax to the mitochondria, which triggers cell apoptosis (8).

CyclinA2 is a highly conserved protein that is encoded by the CCNA2 gene (9). CyclinA2 combined with cyclin-dependent kinase (CDK) 1 and CDK2 controls the transition of the cell cycle from the G_1_/S phase to the G_2_/M phase and promotes cell mitosis. In general, CyclinA2 is silenced in postnatal hearts (10). CyclinA2 plays a crucial role in cardiomyocyte growth in fetal and neonatal hearts, and artificially continued expression of CyclinA2 in adult hearts induces cardiomyocyte proliferation and/or hyperplasia (10). Therapeutic delivery of CyclinA2 into adult rat hearts has also been observed to induce cardiomyocyte regeneration following myocardial ischemia (11). To date, the effect of CyclinA2 on cardiomyocyte growth in *in vitro* hypoxic conditions has not been examined; this is therefore the focus of the present study.

## Materials and methods

### Materials

Sprague Dawley neonatal rats were obtained from the Animal Center of Xinxiang Medical University (Xinxiang, China). The study and animal use were approved by the Ethics Committee of Xinxiang Medical University. Dulbecco’s modified Eagle’s medium (DMEM) was purchased from Gibco-BRL (Grand Island, NY, USA). Fetal bovine serum (FBS) and a cell counting kit-8 (CCK-8) were purchased from Hangzhou Sijiqing Bioengineering Material Co., Ltd. (Hangzhou, China). Enhanced green fluorescent protein (EGFP)-adenovirus capsids with and without CyclinA2 cDNA were obtained from Shanghai Genechem Co., Ltd. (Shanghai, China). Rabbit anti-rat CyclinA2 and mouse anti-rat β-actin primary antibodies and horseradish peroxidase (HRP)-conjugated secondary antibodies were purchased from Santa Cruz Biotechnology, Inc. (Santa Cruz, CA, USA). Total protein extraction and bicinchoninic acid (BCA) protein analysis kits were purchased from Pierce (Thermo Fisher Scientific, Inc., Rockford, IL, USA). Polyvinylidene fluoride (PVDF) membranes, trypsin, 4-(2-hydroxyethyl)-1-piperazineethanesulphonic acid, tetramethylethylenediamine and EDTA were purchased from Sigma-Aldrich (St. Louis, MO, USA). Centrifugal filter units were purchased from Millipore Corporation (Billerica, MA, USA).

### Cell culture and treatments

Cardiomyocytes were isolated from neonatal rat hearts as previously described and cultured in the DMEM supplemented with 20% FBS (12). The cells were randomly separated into six groups: Control, hypoxia, EGFP-Adv, EGFP-Ccna2, EGFP-Adv + hypoxia, and EGFP-Ccna2 + hypoxia. The cells in the control group were cultured in a general cell incubator; those in the EGFP-Adv group were transfected with EGFP-adenovirus capsids for 18 h, and then placed in a cell incubator for an additional 12 h; those in the EGFP-Ccna2 group were transfected with EGFP-adenovirus capsids with CyclinA2 cDNA for 18 h, and then placed in a cell incubator for an additional 12 h; those in the EGFP-Adv + hypoxia group were transfected with EGFP-adenovirus capsids for 18 h, and then placed in a hypoxia chamber for an additional 12 h; and those in the EGFP-Ccna2 + hypoxia group were transfected with EGFP-adenovirus capsids with CyclinA2 cDNA for 18 h, and then placed in a hypoxia chamber for an additional 12 h.

### Adenovirus transfection

Cardiomyocytes were plated into 24- and six-well plates and cultured with complete growth medium for 48 h. The complete medium was then replaced with fresh DMEM without serum and antibiotics, and the cells were cultured for an additional 12 h. The cells were subsequently transfected with EGFP-adenovirus capsids [1×10^9^ plaque-forming units (PFU)] or EGFP-adenovirus capsids with CyclinA2 cDNA (1×10^9^ PFU) for 18 h. GFP fluorescence indicated the transfection efficiency.

### Exposure to hypoxia

The cardiomyocytes in the hypoxia, EGFP-Adv + hypoxia and EGFP-Ccna2 + hypoxia groups were placed into a special hypoxia chamber. The chamber was tightly closed and the air was fully replaced with N_2_ three times. The chamber was then placed in a 37°C incubator for 12 h.

### Immunochemical staining

Following the appropriate treatment, the cardiomyocytes grown on the coverslips were fixed with buffered paraformaldehyde for 15 min, treated with 0.1% Triton X-100 in phosphate-buffered saline (PBS) for 10 min, and then incubated with 2% H_2_O_2_ for 1 h at room temperature. Following incubation, the cells were further incubated with 10% goat serum/1% bovine serum albumin (BSA) in PBS at room temperature for 30 min, and then incubated with CyclinA2 primary antibody (1:200 in 1% BSA) at 4°C overnight. Subsequent to washing with PBS, the cells were incubated with HRP-conjugated secondary antibody (1:500 in 1% BSA) at room temperature for 1 h. The cells were then incubated with 3,3′-diaminobenzidine substrate at room temperature for 5 min. The images were viewed and captured using a BH-2 Olympus microscope (Olympus Corporation, Tokyo, Japan).

### Western blot analysis

Proteins were extracted from the treated cells using a total protein extraction kit and the protein concentrations were measured using a BCA protein analysis kit. Proteins (20 μg/sample) were subsequently separated by SDS-PAGE, and then transferred to the PVDF membranes. The membranes were incubated with 5% non-fat milk in Tris buffered saline with Tween-20 (TBST) for 1 h, and then incubated with CyclinA2 or β-actin primary antibodies (1:1,000) at 4°C overnight. The membranes were then incubated with HRP-conjugated second antibody (1:10,000) for 1 h at room temperature. The immunoreactive bands were visualized by enhanced chemiluminescence.

### Cell viability assay

Following an 18-h transfection, cardiomyocytes (5×10^3^/well) were plated in 96-well plates. In parallel, another portion of cells (non-transfected) were directly plated in the 96-well plates, serving as samples in the control and hypoxia groups. The cells in the hypoxia, EGFP-Adv + hypoxia and EGFP-Ccna2 + hypoxia groups were kept in a hypoxia chamber for 12 h at 37°C, and the cells in the control, EGFP-Adv, EGFP-Ccna2 groups were cultured in a general cell culture incubator for 12 h. Cell viability was then measured using a CCK-8 in accordance with the manufacturer’s instructions (cat. no. C0037; Hangzhou Sijiqing Bioengineering Material Co., Ltd.). The absorbance was read using a plate reader at 540 nm.

### Statistical analysis

Statistical analysis was performed using SPSS 16.0 software (SPSS, Inc., Chicago, IL, USA). Data are presented as the mean ± standard error. Univariate comparisons of means were evaluated using one-way analysis of variance with Tukey’s post hoc adjustment for multiple comparisons. P<0.05 was considered to indicate a statistically significant difference.

## Results

### Morphology of primary cardiomyocytes

**Figure 1:**
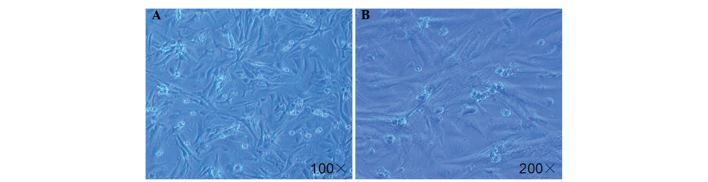
Morphology of primary cardiomyocytes isolated from neonatal rats following plating for five days.

As shown in Fig. 1, the primary cardiomyocytes from the neonatal rat hearts reached 80% confluence subsequent to plating for five days. The majority of the cells (>90%) were rod-shaped myocytes, while a few cells (<10%) were round-shaped non-myocytes. These data are consistent with the results from a previous study (13).

### Efficiency of adenovirus transfection

**Figure 2:**
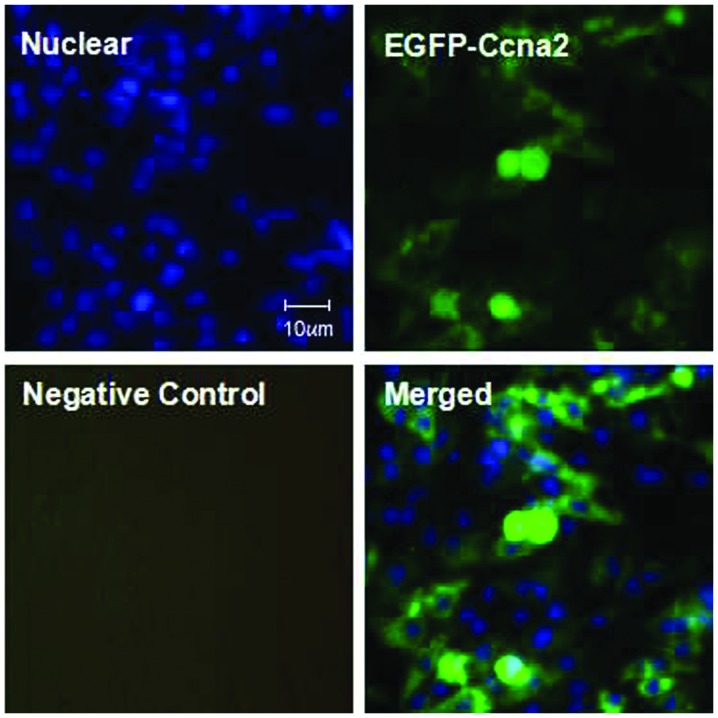
GFP fluorescence (green fluorescence) indicates transfection efficiency [green positive cells versus blue positive (total) cells] following incubation of the cardiomyocytes with EGFP-adenovirus capsids with CyclinA2 cDNA (EGFP-Ccna2) for 18 h (magnification, ×200). DAPI staining (blue fluorescence) indicates the nucleus. EGFP, enhanced green fluorescent protein.

In this study, the transfection efficiency was indicated by the positive rates of GFP fluorescence (green). As shown in Fig. 2, following the transfection of the EGFP-adenovirus capsids with CyclinA2 cDNA for 18 h, >80% of the cardiomyocytes were positive for green fluorescence (GFP), which suggested that the CyclinA2 gene was also overexpressed in the majority of the cells following the transfection.

### Expression levels of CyclinA2 following treatments

**Figure 3:**
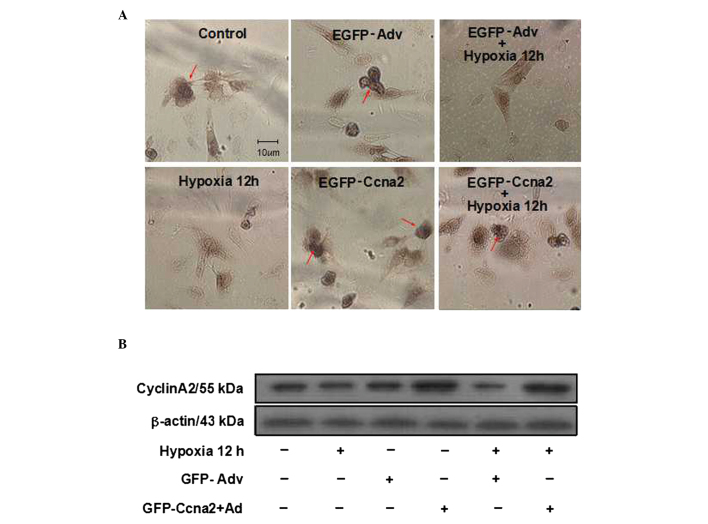
Expression of CyclinA2 following the different treatments. (A) Immunochemical staining showing CyclinA2 expression in each group (red arrows indicate CyclinA2 distribution in the nucleus) (magnification, ×400); (B) western blot analysis showing CyclinA2 expression in each group. EGFP, enhanced green fluorescent protein.

In this study, protein expression of CyclinA2 following the different treatments was measured by immumochemical staining and western blot analysis. Consistent with a previous study (14), the results showed that CyclinA2 was mainly distributed in the nucleus (Fig. 3A). Immunochemical staining and western blot analysis showed that CyclinA2 expression was markedly downregulated in the hypoxia group as compared with that in the control group (Fig. 3A and B). Compared with the transfection of EGFP-adenovirus capsids (the EGFP-Adv group), CyclinA2 expression was markedly increased following the transfection of EGFP-adenovirus capsids with CyclinA2 cDNA (the EGFP-Ccna2 group). Furthermore, CyclinA2 expression in the EGFP-Ccna2 + hypoxia group was also much higher than that in the EGFP-Adv + hypoxia group (Fig. 3A and B).

### Effect of CyclinA2 overexpression on cardiomyocyte proliferation

**Figure 4:**
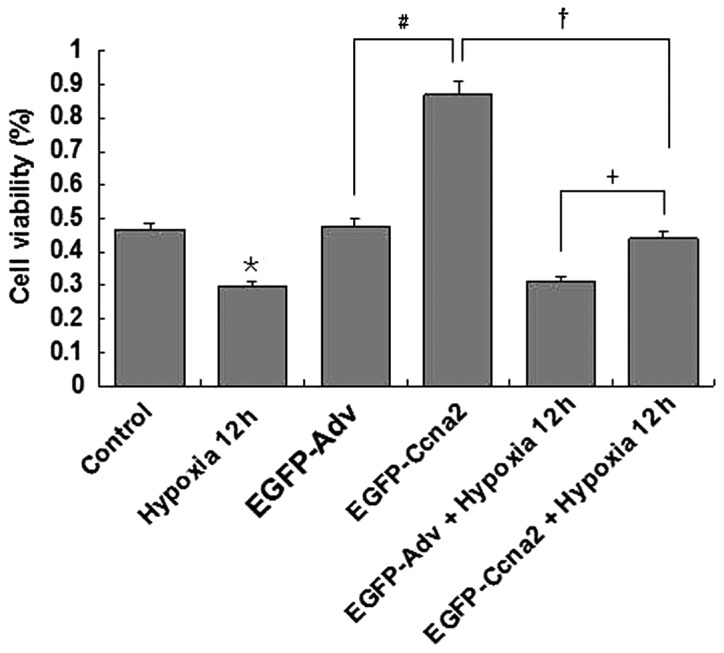
Effect of CyclinA2 overexpression on cardiomyocyte proliferation in hypoxic conditions. Data are presented as the mean ± standard error (n=6 per group). ^*^P<0.05 vs. the control group; ^#^P<0.05 vs. the EGFP-Adv group; ^+^P<0.05 vs. the EGFP-Adv + hypoxia group; ^†^P<0.05 vs. the EGFP-Ccna2 group. EGFP, enhanced green fluorescent protein.

In this study, cardiomyocyte proliferation was measured using a CCK-8 kit. As shown in Fig. 4, hypoxic treatment (12 h) markedly decreased the proliferation of cardiomyocytes (P<0.05, hypoxia group versus control group). Overexpression of CyclinA2 significantly increased cardiomyocyte proliferation (P<0.05, EGFP-Ccna2 group vs. EGFP-Adv group). Hypoxic treatment also markedly decreased the proliferation of cardiomyocytes overexpressing CyclinA2 (P<0.05, EGFP-Ccna2 + hypoxia group versus EGFP-Ccna2 group); however, CyclinA2 overexpression partially recovered the hypoxia-impaired proliferation of cardiomyocytes (P<0.05, EGFP-Ccna2 + hypoxia group versus EGFP-Adv + hypoxia group).

## Discussion

The results of the present study demonstrate that hypoxia inhibits the proliferation of cardiomyocytes isolated from neonatal rat hearts, and overexpression of CyclinA2 partially recovers cardiomyocyte proliferation in hypoxic conditions. Furthermore, CyclinA2 overexpression increases cardiomyocyte proliferation in normal culture conditions. These findings suggest that CyclinA2 plays a key role in the regulation of cardiomyocyte growth, and its upregulation protects cardiomyocytes from the loss of their proliferative ability in hypoxic conditions.

HF has become a leading cause of mortality and morbidity in the world. HF is usually caused by acute myocardial infarction following heart ischemia. While prolonged ischemia without reperfusion causes the direct death of cardiomyocytes, ischemia with reperfusion also leads to myocardial loss via free radical-induced cardiomyocyte apoptosis (3,15). It is known that mammalian cardiomyocytes are terminal and non-proliferative cells. The myocardial injury is generally repaired by fibroblast proliferation and ECM accumulation in adults (16). This massive ECM accumulation leads to cardiac fibrosis, dysfunction and even failure (17).

CyclinA2 is a highly conserved protein that is involved in control of the transition of the cell cycle from the G_1_/S phase to the G_2_/M phase, and cell mitosis (18). In mammals, cardiomyocytes generally lose their proliferative ability a few days after birth, which is associated with a sharp downregulation of CyclinA2 in the hearts following the birth (10). Previous studies have shown that CyclinA2 is nearly silenced in adult hearts, and delivery of CyclinA2 to adult animal hearts can partially recover cardiomyocyte growth (10,11). In the present study, it was observed that overexpression of CyclinA2 increased the proliferation of primary neonatal rat cardiomyocytes. Furthermore, it was shown that CyclinA2 was markedly downregulated in the neonatal rat cardiomyocytes following exposure to hypoxia for 12 h, and cardiomyocyte proliferation was also markedly decreased following the hypoxic treatment. Of note, overexpression of CyclinA2 was able to partially recover the hypoxia-impaired cardiomyocyte growth.

In conclusion, this study clearly demonstrates that overexpression of CyclinA2 in neonatal cardiomyocytes cultured *in vitro* can promote their proliferation in physiological (general culture) and pathophysiological (hypoxic exposure) conditions. The data obtained from this study will provide further support for the application of CyclinA2 for the induction of myocardial regeneration.
